# A reinvestigation of recruitment to randomised, controlled, multicenter trials: a review of trials funded by two UK funding agencies

**DOI:** 10.1186/1745-6215-14-166

**Published:** 2013-06-09

**Authors:** Ben G O Sully, Steven A Julious, Jon Nicholl

**Affiliations:** 1 Medical Statistics Group, School of Health and Related Research, University of Sheffield, 30 Regent Court, Regent Street, Sheffield S1 4DA, UK; 2 School of Health and Related Research, University of Sheffield, 30 Regent Court, Regent Street, Sheffield S1 4DA, UK

**Keywords:** Extension, Health Technology Assessment, Medical Research Council, Power, Recruitment, Sample size

## Abstract

**Background:**

Randomised controlled trials (RCTs) are the gold standard assessment for health technologies. A key aspect of the design of any clinical trial is the target sample size. However, many publicly-funded trials fail to reach their target sample size. This study seeks to assess the current state of recruitment success and grant extensions in trials funded by the Health Technology Assessment (HTA) program and the UK Medical Research Council (MRC).

**Methods:**

Data were gathered from two sources: the National Institute for Health Research (NIHR) HTA Journal Archive and the MRC subset of the International Standard Randomised Controlled Trial Number (ISRCTN) register. A total of 440 trials recruiting between 2002 and 2008 were assessed for eligibility, of which 73 met the inclusion criteria. Where data were unavailable from the reports, members of the trial team were contacted to ensure completeness.

**Results:**

Over half (55%) of trials recruited their originally specified target sample size, with over three-quarters (78%) recruiting 80% of their target. There was no evidence of this improving over the time of the assessment. Nearly half (45%) of trials received an extension of some kind. Those that did were no more likely to successfully recruit. Trials with 80% power were less likely to successfully recruit compared to studies with 90% power.

**Conclusions:**

While recruitment appears to have improved since 1994 to 2002, publicly-funded trials in the UK still struggle to recruit to their target sample size, and both time and financial extensions are often requested. Strategies to cope with such problems should be more widely applied. It is recommended that where possible studies are planned with 90% power.

## Background

This study seeks to assess the current state of recruitment success and grant extensions in trials funded by the UK Health Technology Assessment (HTA) program and the UK Medical Research Council (MRC). This work updates a review for the interval 1994 to 2002 by McDonald et al. [1].

When planning a trial, one essential step is the calculation of a sample size that will give the minimum number of participants required to meet the objectives of the study [2].

Having a good estimate of the sample size is important, as studies that are either too small or too large may be judged unethical [3]. For example, a study that is too large could have met the objectives of the trial before the actual study end had been reached, meaning that some patients may have unnecessarily entered into the trial and been randomised to a therapy that can be proven to be suboptimal. Conversely, a trial that is too small may have little chance of meeting the study objectives, and patients may be entering a trial for no tangible benefit.

Poor recruitment is acknowledged as an important shortcoming of many randomised controlled trials (RCTs), which can prevent a study from reaching its target sample size [1,4,5]. Of trials published in the *British Medical Journal* (*BMJ*) and the *Lancet* between 2000 and 2001, 51% of multicentered trials reported difficulties in recruitment [6], while a 2006 review of publicly-funded multicenter trials found that less than a third of all studies achieved their recruitment target and half of all studies received an extension [1].

The consequences of poor recruitment are varied. If the target sample size is not met then the chances of seeing a statistically significant result when there is a true difference between treatments will be reduced, therefore decreasing the likelihood of finding evidence of an effect for a particular health technology.

Poor recruitment also has a negative impact on a trial’s costs: if an extension is required to obtain the target sample size, the trial’s budget will be increased.

While the issue is not new, there is little quantitative research on the extent of the problem. This review aims to update research funded by the UK National Health Service (NHS) R&D National Methodology Programme and the UK MRC in 2006, which looked at recruitment to publicly-funded multicenter trials between 1994 and 2002 [1]. In this study, we update this review to look at studies undertaken between 2002 and 2008.

## Methods

### Trial identification

We looked at 73 trials funded by the HTA and the UK MRC. Data were collected from the online databases held by the bodies: the MRC subset of the Current Controlled Trials metaregister (http://www.controlled-trials.com/mrct/), and the HTA archive of published articles (http://www.hta.ac.uk/project/htapubs.asp). Inclusion and exclusion criteria were chosen to match the previous study [1]. Trials were eligible if: (1) they were multicenter (multicenter trials are commonly reported to recruit more slowly than expected [7]), (2) recruitment started on or after 1 January 2002, (3) recruitment was originally planned to close on or before 31 December 2008 (trials that were awarded an extension beyond 31 December 2008 were included if they had closed to recruitment when data were extracted), and (4) they were not a cluster randomised trial (these were excluded because recruitment issues differ).

These criteria were chosen to be consistent with McDonald et al. [1] to ensure comparability. Note that there is no overlap between these two pieces of research: the previous study required trials to have finished recruiting by 1 January 2002, and ours requires trials to have started recruiting on or after this period.

### Data extraction

The International Standard Randomised Controlled Trial Number (ISRCTN) was captured for each trial. For HTA-funded trials, the original HTA publication was used. MRC-funded trials were investigated on a case-by-case basis, where possible using the original study protocol, clinical report, or trial website found by using the ISRCTN. For trials where no information could be found, the principal investigator or trial manager was contacted.

While the reporting of trials was largely of a good standard, there were limits on the trial details published. Occasionally, therefore, we were not able to gather information on certain specifics, denoted by the ‘Missing’ values in tables.

Trial recruitment was classed as a ‘success’ if the original or revised recruitment target was met, or if the trial was stopped early due to an interim analysis. Trials that were terminated (for example, due to slow recruitment) were classed as failing to recruit.

Where appropriate, *χ*^2^ tests and *χ*^2^ tests for trend were used to test for differences between the two study periods (1994 to 2002 and 2002 to 2008), between trials with and without a clinical trials unit (CTU), and between trials that successfully recruited and those that did not. Categories with insufficient data and categories corresponding to missing data were excluded from these tests. Data were collected and analyzed in SPSS version 19 (SPSS Inc., Chicago, IL, USA).

### Power calculations

In addition to capturing recruitment, the planned power for each trial was also captured. Using this along with the target and achieved recruitment allows the true achieved power to be calculated for each trial. This will be performed by simply rearranging and applying the appropriate sample size formula using the proportion of the target sample size that was actually recruited, and does not require using any other trial results. The process for each study is as follows:

1. Assume a planned type I error *α* of 0.05, and use the planned sample size *n* and the planned power *1-β* to determine the approximate standardised effect size δ of the study using the formula:

$$\delta^{2}=2\left(Z_{1-\alpha /2}+Z_{1-\beta }\right)$$

2. Calculate the proportion *π* of the planned sample size *n* actually recruited to the study

3. Calculate the achieved power of a study with parameters *α, δ*^*2*^ and *σ*^*2*^ and sample size *πn* using the formula:

$$\left(1-\beta \right)\mathrm{achieved}=\Phi \left[\sqrt{\frac{\mathrm{\pi n}\delta^{2}}{2}}-Z_{1-\alpha /2}\right]$$

## Results

**Figure 1:**
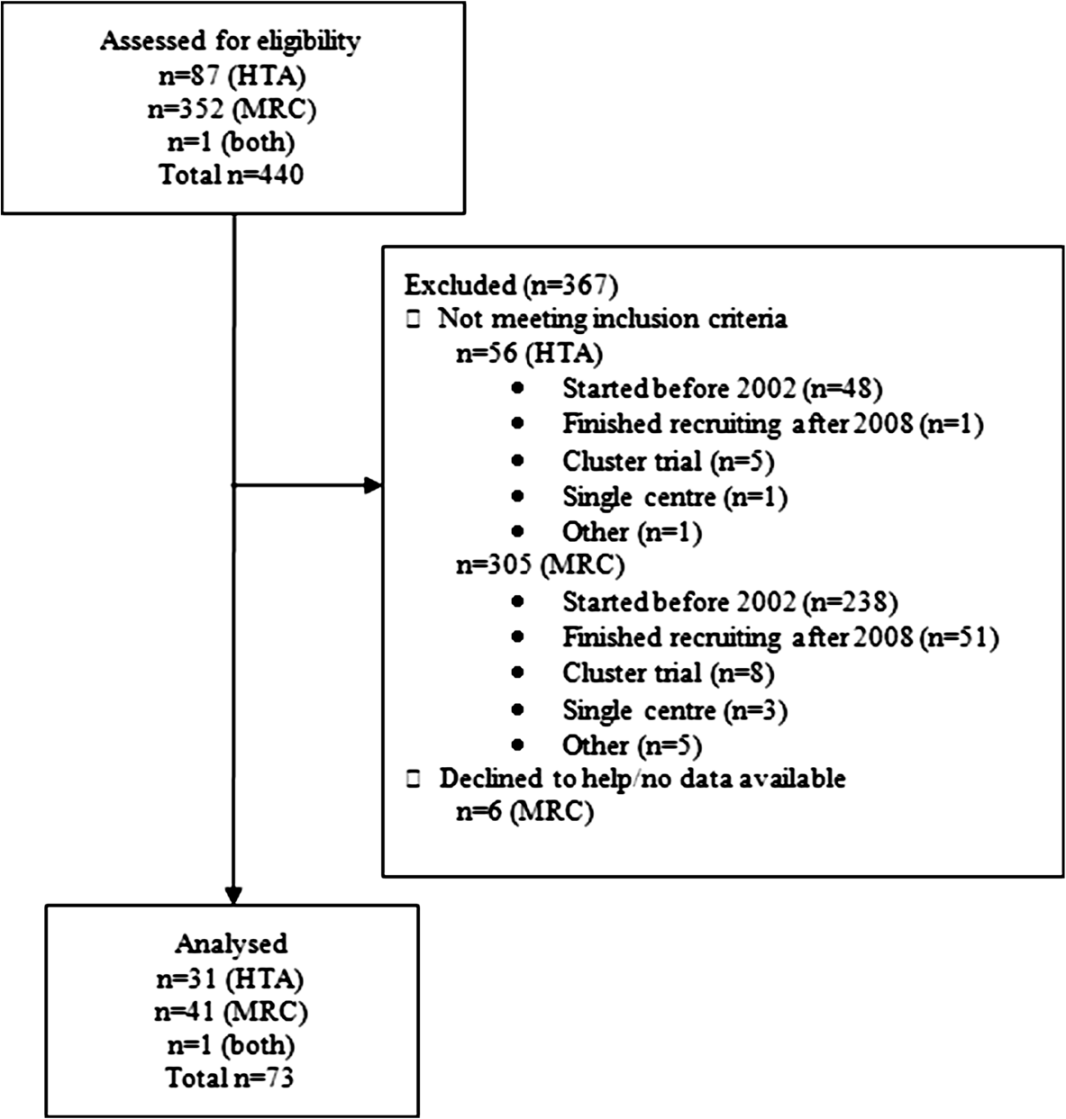
Flow diagram for the study.

The databases contained 73 trials fulfilling the inclusion criteria. Figure 1 shows the flow of trials through the review; 31 (43%) were funded by the HTA, 41 (56%) by the MRC, and 1 (1%) by both bodies.

### Trial characteristics

Table 1 summarises the individual characteristics of the trials. The majority were simple parallel group trials (66 (90%)) with the remainder being factorial designs (4 (5%)) or partially randomised patient preference designs (3 (4%)). Approximately three-quarters of trials had 2 arms (53, (73%)).

**Table 1:** Characteristics of trials

**Characteristic**	**1994 to 2002**		**2002 to 2008**	
	**N**	**n (%)**	**N**	**n (%)**
What type of trial design was used?	122		73	
Parallel		113 (93)		66 (90)
Factorial		6 (5)		4 (5)
Partially randomised patient preference		3 (2)		3 (4)
How many arms were there in the trial?	122		73	
Two		94 (77)		53 (73)
Three		18 (15)		14 (19)
More than three		10 (8)		6 (8)
What clinical area was the trial investigating?	122		73	
Cancer		25 (20)		4 (5)
Mental health (including neurosciences/psychiatry/psychology)		21 (17)		13 (18)
Orthopedics/rheumatology (including back pain)		21 (17)		1 (1)
Obstetrics and gynecology		9 (7)		2 (3)
Primary care		8 (7)		13 (18)
Cardiology		5 (4)		4 (5)
Gastroenterology		5 (4)		0 (0)
Incontinence/urology		5 (4)		3 (4)
HIV/AIDS		5 (4)		4 (5)
Other		18 (15)		29 (40)
In what setting did the trial take place?	122		73	
Hospital		64 (53)		29 (40)
General practice		26 (21)		12 (16)
Mixed		16 (13)		17 (23)
Community		7 (6)		15 (21)
Missing		9 (7)		0 (0)
Were there any recruiting centers outside the UK?	114		73	
No		88 (77)		62 (85)
Yes		25 (22)		11 (15)
Missing		1 (1)		0 (0)
Was a clinical trials unit involved?	114		73	
Yes		89 (78)		31 (42)
No		25 (22)		40 (55)
Missing		0 (0)		2 (3)
Which body funded the trial?	122		73	
UK Medical Research Council (MRC)		73 (64)		41 (56)
UK Health Technology Assessment (HTA)		41 (36)		31 (43)
Both		0 (0)		1 (100)

The most common clinical areas for the trials were in mental health (13 (18%)) and primary care (13 (18%)), which is a slight change from previous findings [1] where cancer and orthopedics/rheumatology trials were the most prevalent. There was a large variety in the clinical area being investigated, reflected by the large number of clinical areas labeled as ‘Other’. We also found a slight decrease in the number of hospital-based trials (40% vs 53% reported previously [1]) with more community-based trials (21%, up from 6%).

### Recruitment

Target recruitments ranged from 56 to 8,000 participants, whereas actual recruitment ranged from 44 to 8,164 participants. Information on the distribution of both target and achieved sample sizes of trials is shown in Table 2.

**Table 2:** Numerical characteristics of trials

**Characteristic**	**Valid n (%)**	**Recruited successfully (%)**	**Mean (SD)**	**Median**	**Minimum to maximum**
No. of centers:					
Total	60 (82)	34 (57)	38.6 (47.9)	15.5	2 to 205
2 to 5	16 (27)	9 (56)	3.4 (1.3)	3.5	
6 to 10	9 (15)	5 (56)	7.6 (1.4)	8.0	
11 to 20	7 (12)	4 (57)	14.3 (2.7)	13.0	
21 to 50	11 (18)	6 (55)	33.2 (8.4)	34.0	
51 to 100	9 (15)	4 (44)	68 (12.6)	68.0	
101+	8 (13)	6 (75)	139.5 (39.5)	119.0	
Target recruitment:					
Total	73	40 (55)	706.3 (1,098.0)	388.0	56 to 8,000
1 to 100	6 (8)	4 (67)	77.8 (14.1)	78.0	
101 to 200	11 (15)	6 (55)	156.8 (30.4)	150.0	
201 to 400	24 (33)	15 (63)	315.9 (56.6)	305.0	
401 to 1,000	22 (30)	10 (46)	637.5 (168.5)	595.0	
1,001+	10 (14)	5 (50)	2,776 (1,938.7)	2,250.0	
Final recruitment					
Total	73	40 (55)	623.7 (1,117.3)	325.0	44 to 8,164
1 to 100	7 (10)	1 (14)	80.3 (36.8)	58.0	
101 to 200	14 (19)	8 (57)	160.4 (60.1)	151.0	
201 to 400	24 (33)	14 (58)	295.8 (75.6)	284.0	
401 to 1,000	20 (27)	12 (60)	519.2 (313.9)	538.5	
1,001+	8 (11)	5 (63)	2,476.3 (2,269.6)	2,478.5	

Table 3 describes the success of trial recruitment in comparison to their original targets. The target was only met in 40 (55%) of trials; meanwhile, 17 (23%) trials recruited to 80% but less than 100% of their target. This is an improvement compared to 1994 to 2002, when only 31% of trials successfully recruited their target (*P* value associated with this difference = 0.002). We also found that fewer trials revised their sample size than in 1994 to 2002, with only 19% doing so (down from 34%; *P* = 0.036). In five (36%) of these, the target was revised upwards; the remaining nine (64%) of trials revised the target downwards. The new target was met in the majority (10 (71%)) of trials in which it was revised, another improvement since the previous study period (19 (45%)).

**Table 3:** Recruitment in trials

	**1994 to 2002**		**2002 to 2008**							
	**Total**		**Total**		***P*value**	**With CTU**		**Without CTU**		***P*value**
	**N**	**n (%)**	**N**	**n (%)**		**N**	**n (%)**	**N**	**n (%)**	
Was recruitment a success?	122		73		0.002^a^	31		40		0.235^a^
Yes		38 (31)		40 (55)			20 (65)		19 (48)	
No		84 (69)		33 (45)			11 (36)		21 (53)	
Was the recruitment target revised?	122		73		0.036^a^	31		40		0.1183^a^
Yes		42 (34)		14 (19)			3 (10)		11 (28)	
No		76 (62)		56 (77)			27 (87)		28 (70)	
Missing		4 (3)		3 (4)			1 (3)		1 (3)	
Final recruitment figure										
Original target:	122		73		<0.001^b^	31		40		<0.001^b^
≥100%		38 (31)		40 (55)			20 (65)		19 (48)	
≥80% but <100%		29 (24)		17 (23)			8 (26)		9 (23)	
<80%		55 (45)		16 (22)			3 (10)		12 (30)	
Revised target:	42		14		0.021^b^	3		11		N/A^b^
≥100%		19 (45)		10 (71)			2 (67)		8 (73)	
≥80% but <100%		15 (36)		3 (21)			0 (0)		3 (27)	
<80%		8 (19)		1 (7)			1 (33)		0 (0)	

^a^*P* values calculated using *χ*^2^ tests, excluding missing categories.

^b^*P* values calculated using *χ*^2^ tests for trend; in the final case counts were too small to perform tests.

CTU, clinical trials unit; N/A, not applicable.

The planned size of a trial appears to have a slight negative effect on the success of recruitment, but the effect is less pronounced than may be imagined: trials requiring over 1,000 participants recruit successfully 50% of the time, compared to trials requiring 100 to 200 participants 55% of the time, and 1 to 100 people 67% of the time. Some categories also contained few studies, so may not be representative of the success of all trials.

The impact of clinical trials units (CTUs) on trials is positive. A total of 31 (42%) trials had CTUs involved, down from 78% in 1994 to 2002 [3]. Of these 31, 20 (65%) recruited successfully, while trials without CTUs successfully recruited only 48% of the time (19 trials).

Table 4 shows how other trial characteristics affected the recruitment of trials. MRC-funded trials appear to recruit successfully more often than HTA-funded trials (25 (61%) compared to 14 (45%)) although the difference is not statistically significant (*P* = 0.270). The clinical area of a trial, however, does seem to influence recruitment success: eight (62%) mental health trials recruited successfully compared to only three (23%) primary care trials. However, the sample size for the majority of these categories is too small to perform meaningful statistical tests. Trials with ≤16 centers (the median number of centers) did not recruit any more successfully than those with >16 centers (57% vs 57%).

**Table 4:** How factors affected recruitment

**Factor**	**Was recruitment a success?**		
	**Yes, n (%)**	**No**, **n (%)**	***P*value**
Funding body:			0.182^a,b^
UK Medical Research Council (MRC)	25 (61)	16 (39)	
UK Health Technology Assessment (HTA)	14 (45)	17 (55)	
Both	1 (100)	0 (0)	
Setting:			0.970^a^
Hospital	16 (55)	13 (45)	
General practice	6 (50)	6 (50)	
Mixed	10 (59)	7 (41)	
Community	8 (53)	7 (47)	
Clinical area:			
Cancer	3 (75)	1 (25)	
Mental health (including neurosciences/psychiatry/psychology)	8 (62)	5 (39)	
Orthopedics/rheumatology (including back pain)	1 (100)	0 (0)	
Obstetrics and gynecology	1 (50)	1 (50)	
Primary care	3 (23)	10 (77)	
Cardiology	1 (25)	3 (75)	
Incontinence/urology	3 (100)	0 (0)	
HIV/AIDS	3 (75)	1 (25)	
Other	17 (59)	12 (41)	
Number of centers:			
≤16	17 (57)	13 (43)	
>16	17 (57)	13 (43)	
Missing	6	7	

^a^*P* values calculated using *χ*^2^ tests without continuity corrections.

^b^‘Both’ column excluded from *χ*^2^ test due to low counts.

Three (4%) trials had unscheduled trial terminations. One was halted for ethical reasons discovered during the trial (another study showing negative results was published during the trial and the intervention was deemed to be ineffective; at this point the target sample size had not been reached) and two due to slow recruitment. These were all coded as being unsuccessful in recruitment.

### Extensions

Table 5 summarises the number of extensions given to trials. Just over half (39 (53%)) of trials had no extension. Those that did largely received time (22 (30%)) or time and grant (10 (14%)) extensions. Only a single (1%) trial received a grant extension alone. These data were missing for one (1%) trial. A total of 20 (64%) trials funded by the HTA required an extension of some kind, compared to only 12 (29%) of trials funded by the MRC, a statistically significant difference (*P* <0.01).

**Table 5:** Extensions to trials

**Was an extension granted and if so**, **what type?**	**1994 to 2002**		**2002 to 2008**									
	**Total**		**Total**		**With CTU**		**Without CTU**		**HTA**		**MRC**	
	**N**	**n (%)**	**N**	**n (%)**	**N**	**n (%)**	**N**	**n (%)**	**N**	**n (%)**	**N**	**n (%)**
	122		73		31		40		31		41	
No		57 (47)		39 (53)		13 (42)		25 (63)		11 (36)		28 (68)
Time extension		15 (12)		22 (30)		14 (45)		7 (18)		17 (55)		4 (10)
Grant extension		8 (7)		1 (1)		0 (0)		1 (3)		0 (0)		1 (2)
Time + grant extension		42 (34)		10 (14)		4 (13)		6 (15)		3 (10)		7 (17)
Missing		0 (0)		1 (1)		0 (0)		1 (3)		0 (0)		1 (2)

CTU, clinical trials units; HTA, Health Technology Assessment; MRC, Medical Research Council.

Not all studies reported whether they received an extension. Where an extension was not reported we contacted the trial’s chief investigator as identified through the ISRCTN database; this was necessary for eight trials, and in one case we did not get a reply. This was coded as missing in the database.

**Figure 2:**
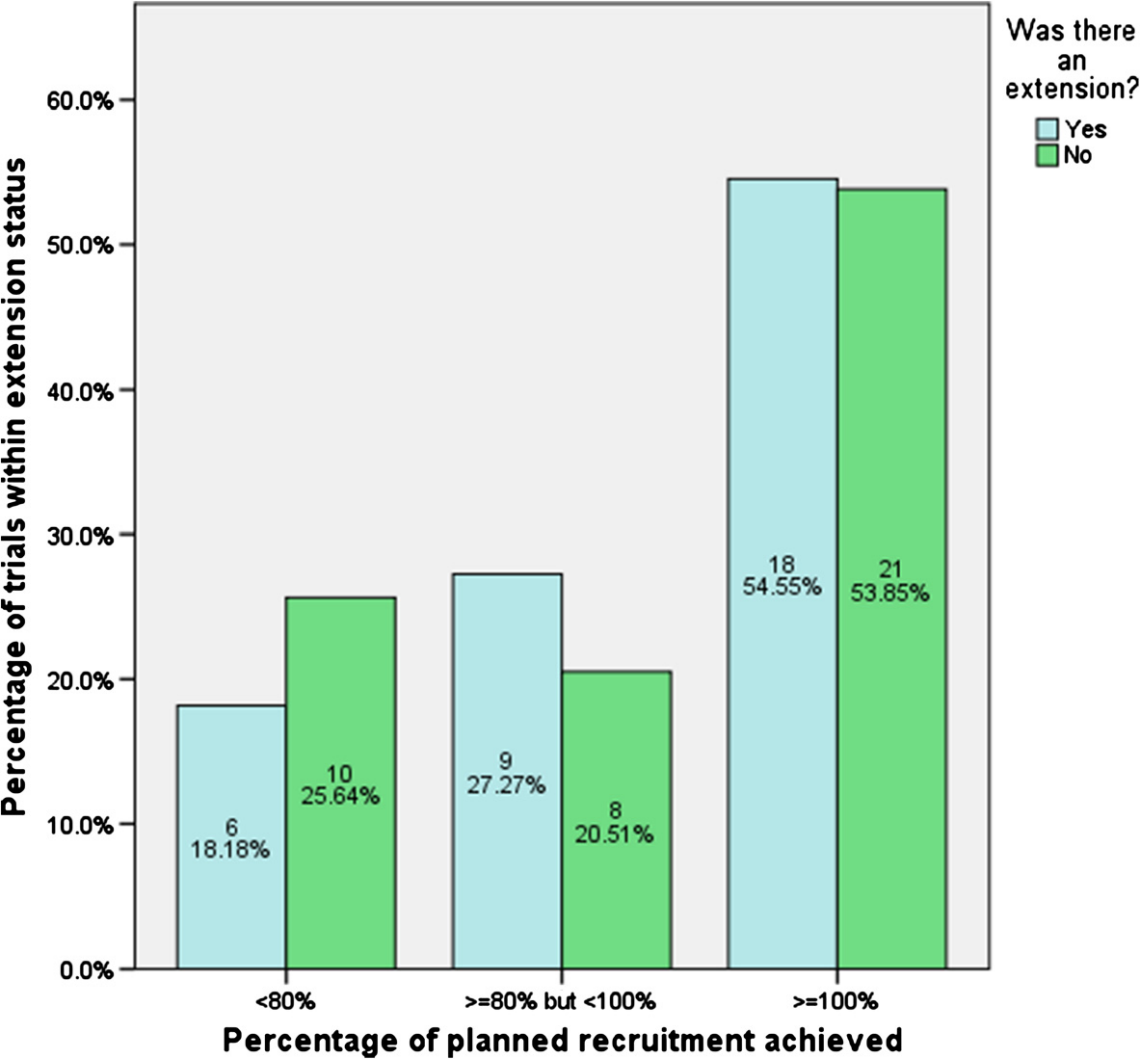
Recruitment by extension status.

These data are simplified in Figure 2, where all extensions have been grouped together. In all, 33 (45%) trials received an extension and of these 18 (55%) recruited to their target. A total of 39 (53%) trials did not receive an extension; of these, 21 (54%) recruited successfully. Hence, trials receiving extensions are equally likely to recruit to 100% of their target sample size as those not receiving extensions; however, they are more likely to recruit at least 80% of their target (see Figure 2).

### Power

**Figure 3:**
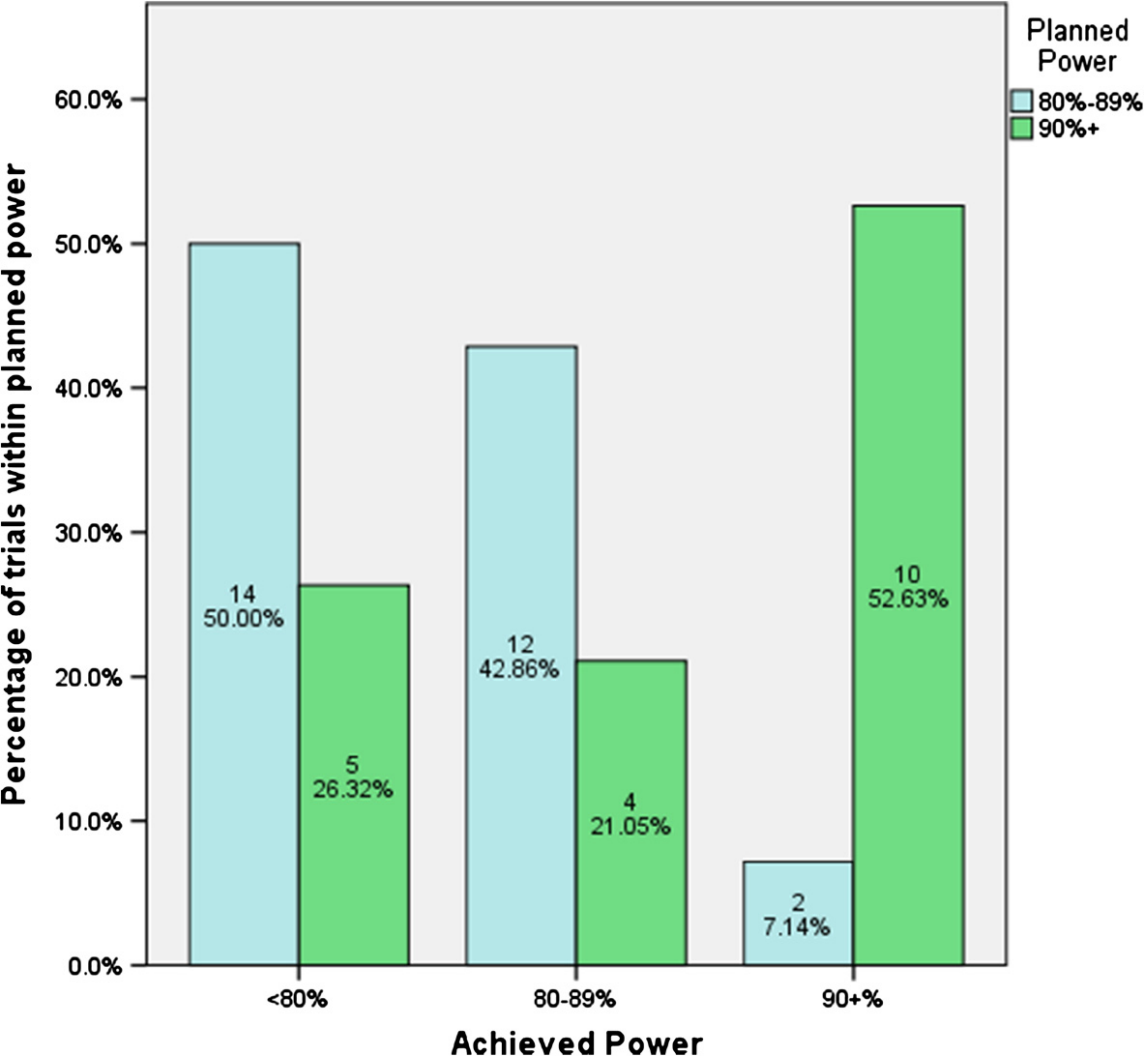
Achieved power of studies by planned power (missing data omitted).

Results from power calculations are summarised in Table 6 and Figure 3. Only three trials prespecified their power as any number other than 80% or 90%; one planned 83%, one 85%, and one 95% power. Of studies that planned for 90% power, 89% (26) managed to recruit a minimum of 80% of their initially planned sample size, compared with only 72% (28) of trials planned with 80% power.

**Table 6:** Planned and true power of trials

	**Planned power**				
	**80%to 89%**		**90%+**		**Missing**
	**N**	**n (%)**	**N**	**n (%)**	**N**
True power	39		28		6
90%+		2 (7)		10 (53)	
80% to 89%		12 (43)		4 (21)	
<80%		14 (50)		5 (26)	
Missing		11		9	
Final recruitment	39		28		6
≥100%		22 (56)		16 (57)	
≥80% but less than 100%		6 (15)		9 (32)	
<80%		11 (28)		3 (11)	

## Discussion

This review has found improved results compared to the 2006 report by McDonald et al. [1]. Slow or inadequate recruitment to publicly-funded multicenter RCTs is still a common problem however, with a large proportion requiring extensions. The previous report looked at trials recruiting between 1994 and 2002; we updated this to look at trials recruiting between 2002 and 2008. We found slightly fewer eligible trials (73, compared to 114; 10.4 per year compared to 12.7 per year).

Over half of trials recruited to 100% of their original target, up from around one-third in the previous 8 years. Additionally the proportion of trials failing to recruit 80% of their target in 2002 to 2008 was around one-fifth; down from 45% in 1994 to 2002. This trend is promising and could reflect the large amount of work aimed at increasing recruitment to clinical trials [8], but the proportion of trials recruiting their target is still considerably less than desirable. More research into both the reasons behind poor recruitment and methods of improving it is needed to help increase this over the next decade.

We found that studies performing sample size calculations based on 90% power were much more likely to reach at least the minimum acceptable level of 80% power than those planned based on 80% power, as shown in Figure 3. Over half of studies with a planned power of between 80% and 90% finished with inadequate power. In addition, studies that plan for 90% power are more likely to complete with at least 80% power than those that plan for 80%. Our advice therefore is to design a trial with as high a power as possible: if the study under-recruits there is the opportunity to reduce the power to at least 80% power.

Extensions have become less commonplace, but are still granted in a large proportion of trials. Figure 2 shows that trials receiving an extension are no more likely to recruit 100% of their target than those not receiving one; they are however more likely to recruit between 80% and 100% of their target. That is not to say that extensions have no use, as extensions more than likely facilitated the recruitment achieved.

The proportion of publicly-funded trials using CTUs has decreased compared to pre-2002. However, the HTA now requires evidence of CTU involvement when funding trials, which could have caused this proportion to have increased since 2008. CTU involvement did not reduce the prevalence of extension requests, but was associated with improved recruitment to trials.

Trials with CTUs appear to obtain extensions more often than trials without (58% compared to 36%). This could be because slow recruitment in CTU studies is due to factors beyond the control of the trial team, or it could be because trials involving CTUs are reported more thoroughly and are less likely to omit the presence of an extension. It appears that the HTA are more willing to provide extensions than the MRC (64% of HTA trials were given an extension of some kind, compared to 32% of MRC trials), perhaps due to the type of trials they tend to fund.

There have been many reviews of methods to improve recruitment to trials, particularly in the last 10 years. Watson and Torgerson [5] undertook a systematic review of RCT trialing methods, with the aim of identifying effective strategies. They found that there were very few such trials, but specific strategies to help recruitment included not blinding, educating clinicians, using culturally specific designs and incentives for participants.

With poor recruitment having such prevalence in current RCTs, more advanced statistical methods should be considered in trial planning and analysis. One particularly attractive and increasingly popular strategy is the use of adaptive designs. Such designs provide opportunities to determine the progress of trials, allowing for reassessment of the assumptions in made in the design of a trial as well as to make an assessment of efficacy and futility. They have the potential to save both time and money [9].

One strength of this investigation is its adherence to the methods undertaken by McDonald et al. [1] in their previous study, which allows for a comparison of the results. However, a limitation of this study is the quality of data. All data were retrieved from online reports of HTA-funded and MRC-funded trials, and as such was not always complete. For example, not all studies reported where they had received an extension. It should be noted though that recruitment rates are specified in the Consolidated Standards of Reporting Trials (CONSORT) statement as being required [10], which facilitated extraction of these data.

In undertaking this study we found two studies that were halted due to a significant finding of efficacy in a planned interim analysis by their data monitoring committee, which were subsequently coded as a successful recruitment. As noted in an earlier section there were also two trials that stopped early due to slow recruitment, and another due to ethical reasons; all three of these were coded as a failure to recruit. However, it is quite possible that the studies halted due to efficacy were still under their recruitment target; similarly that the study stopped due to ethical reasons was recruiting to their target. These trials highlight a difficulty of this research where limited information was available on trials, requiring some discretion on the authors’ part.

## Conclusions

This study was performed as an update to the 2006 review by McDonald et al. [1]. We found that although recruitment rates have improved since 1994 to 2002 they are still low, with only around half recruiting to their target. Additionally extensions are still widely requested (and granted), with almost one-third of trials receiving an extension of some kind (down from around half in 1994 to 2002). Trials that fail to reach recruitment targets are less likely to answer their objectives and so could have their ethics questioned.

There is clearly room for research on both ways of improving recruitment, and ways of monitoring and managing low recruitment. Involving a CTU in a trial appears to play a role in increasing recruitment to trials. We would recommend that adaptive designs that enable reassessment of the trial design assumptions are more commonly undertaken in public-funded trials.

Trials designed with 80% power are over twice as likely not to recruit at least 80% of their target sample size as those designed with 90%; indeed, planning a trial with 80% power could be seen as a marker for a study that may fail to recruit. While the commonly used powers of 80% and 90% are arbitrary, we recommend that where possible trials are designed with higher power; this way, trials can consider sacrificing some power if recruitment is not as successful as expected.

## Abbreviations

CONSORT: Consolidated Standards of Reporting Trials; CTU: clinical trials unit; HTA: Health Technology Assessment; ISRCTN: International Standard Randomised Controlled Trial Number; MRC: Medical Research Council; NHS: National Health Service; NIHR: National Institute for Health Research; RCT: Randomised Controlled Trial.

## Competing interests

The authors declare that they have no competing interests.

## Authors’ contributions

BS carried out the data extraction, analysis and report writing. SAJ conceived of the study, assisted with interpretation of results and reporting. JN assisted in analysis, interpretation and reporting. All authors approved the final draft.
